# Analysis of perioperative risk factors for deep vein thrombosis in patients with femoral and pelvic fractures

**DOI:** 10.1186/s13018-020-02131-5

**Published:** 2020-12-10

**Authors:** Linqin Wu, Bo Cheng

**Affiliations:** 1 Department of Anesthesiology, The First Affiliated Hospital of Chongqing Medical University, Yuzhong District, Chongqing, 400000 China; 2 Department of Anesthesiology, The First Affiliated Hospital of Chongqing Medical University, Yuzhong District, Chongqing, 400000 China

**Keywords:** Deep vein thrombosis, Femoral fractures, Pelvic fractures, Risk factors, Perioperative period

## Abstract

**Objective:**

Clinical characteristics, anticoagulant protocols, and risk factors of deep vein thrombosis (DVT) in patients with femoral and pelvic fractures were analyzed throughout the perioperative period to provide references for early identification and optimization of risk factors.

**Methods:**

This was a retrospective study. A total of 569 patients undergoing surgery of femoral and pelvic fractures from May 2018 to December 2019 were included.

The clinical data including general conditions, trauma, surgery, anticoagulant protocols, and laboratory indexes were collected. According to the results of deep vein Doppler ultrasonography of the lower extremities, the patients were divided into non-DVT group and DVT group. Univariate analysis and multivariate logistic regression analysis were used to identify the independent risk factors of preoperative and postoperative DVT.

**Results:**

The incidence of DVT was 40.25% and preoperative DVT was 26.71%, which was higher than the incidence of postoperative DVT of 17.22%. Most of them were thrombus on the affected side (60.26%) and distal thrombus (81.66%). The average time of DVT formation was 6.55 ± 0.47 days after trauma and 6.67 ± 0.48 days after surgery. Chronic obstructive pulmonary disease (COPD), anemia, hypoproteinemia, non-anticoagulation before surgery, delayed anticoagulation after trauma and admission, high-energy trauma, multiple injuries, drinking history, and advanced age were independent risk factors for perioperative DVT. The increased level of fibrinogen degradation products was an independent risk factor for preoperative DVT. These risk factors were identified to be independently associated with postoperative DVT, including intraoperative blood transfusion, postoperative blood transfusion, pulmonary infection, preoperative non-anticoagulation, postoperative delayed anticoagulation, preoperative waiting time > 7 days, operative time > 2 h, c-reactive protein, fibrinogen level, platelet count 1 day after surgery, c-reactive protein, fibrinogen, and hemoglobin levels 3 days after surgery, comminuted fracture.

**Conclusions:**

At present, anticoagulation and other DVT prevention and treatment programs have not changed the current situation that the incidence of DVT is still high. Through the analysis of the risk factors of DVT throughout the perioperative period, optimizing the perioperative blood transfusion, preoperative lung disease, hypoproteinemia, anemia, inflammation, etc., and surgery as soon as possible after trauma may further reduce its incidence.

## Introduction

Most of the patients with femoral and pelvic fractures are at high risk of venous thromboembolism (VTE) due to hypercoagulable state [1], trauma and surgical injury, prolonged immobilization, and edema of surrounding tissues, for example, the incidence of preoperative DVT and the postoperative DVT in femoral neck fracture was 32% and 56% respectively [2]. Previous studies have focused on the analysis and prevention of DVT risk factors after fracture surgery, and seldom focused on the analysis of risk factors before, during and after the entire perioperative period. For example, the influence of preoperative risk factors including chronic obstructive pulmonary disease, pulmonary infection, hypoproteinemia, and perioperative blood transfusion on the occurrence and development of DVT. Secondly, anticoagulation therapy, early activities, and lower extremity pneumatic therapy are the main measures for the prevention and treatment of DVT. Although the incidence of DVT during the perioperative period of femoral and pelvic fractures has been reduced to a certain extent, it has not changed the current status of the high incidence of DVT. Therefore, routine anticoagulant regimens may not be appropriate for preventing fractures in patients with different thrombotic risks.

Therefore, we further analyzed the clinical characteristics and risk factors of DVT in patients with femoral and pelvic fractures, so as to provide reference for early identification and optimization of risk factors and the development of individual anticoagulation protocols, so as to reduce the incidence of perioperative DVT.

## Materials and methods

This study has been approved by the Ethics Committee of the First Affiliated Hospital of Chongqing Medical University (2019-277) and registered in the World Health Organization International Clinical Trial (ChiCTR2000035103).

### Inclusion and exclusion criteria

Inclusion criteria were patients aged 18 years or older, traumatic fracture, definite diagnosis of femoral and pelvic fractures, surgical treatment, and complete data available in medical records. Exclusion criteria were patients combined with diseases of the blood system or coagulation disorders, long-term use of anticoagulant drugs, vascular surgery, and pregnancy.

### Diagnosis of DVT

After admission, C5-1 linear probes, IU22 system (Philips ATL, Bothell, WA, USA) was used to perform pulse Doppler ultrasound examinations on the lower limbs of the patients. The diagnostic criteria of DVT are incompressibility of vein, filling defect of cavity, and lack of Doppler signal. Routine scanning was performed for the femoral common vein, superficial and deep femoral vein, popliteal vein, posterior and anterior tibial vein, peroneal vein of bilateral lower extremities, and intermuscular vein thrombosis.

### Research methods

From May 2018 to December 2019, a total of 905 patients with lower limb traumatic fractures (tibia, fibula, patella, femur, and pelvic fractures) underwent surgical treatment, of which 67.51% (611/905) were femoral and pelvic fractures. According to the inclusion and exclusion criteria, 569 (93.13%) patients with femoral and pelvic traumatic fractures were included in the study. There were 417 males and 152 females, with an average age of 69.6 ± 19.1 years (20–112 years). The subjects included 249 cases of femoral neck fracture, 166 cases of intertrochanteric fracture, 60 cases of femoral shaft fracture, 67 cases of pelvic fracture, and 27 cases of acetabular fracture. After admission, deep vein Doppler ultrasonography of both lower extremities was performed. According to the examination time and results, the patients were divided into preoperative and postoperative non-DVT groups and DVT groups, in order to analyze the risk factors of preoperative and postoperative DVT. The postoperative DVT group included new thrombus formed after surgery or preoperative thrombus with postoperative progress.

### Data collection

We collected clinical data of patients through the electronic medical record system, anesthesia system. The formation time, location, and thrombosis type of DVT were collected. The distal thrombosis is defined as a thrombus that occurs beyond the popliteal vein; proximal thrombus is a popliteal vein and thrombus near the popliteal vein; both proximal and distal thrombus are mixed thrombus. The basic information of patients such as the age, gender, body mass index (BMI), smoking history, and drinking history were collected. Complications include cerebral infarction, diabetes, hypertension, coronary heart disease, hyperlipidemia, liver disease, kidney disease, lung infections, respiratory failure, pulmonary malignant tumor, chronic obstructive pulmonary disease (COPD), hypoproteinemia, anemia, and malignant tumor. Trauma and surgery-related conditions included traumatic factors (low-energy or high-energy trauma), multiple injuries, shock, fracture site, open fracture, comminuted fracture, barotherapy, time from trauma to hospital, waiting time before surgery (time from trauma to operation), preoperative blood transfusion, operation time, type of anesthesia (general anesthesia or not), method of anesthesia (including general anesthesia for tracheal intubation, epidural anesthesia, nerve block), American Society of Anesthesiologists (ASA) anesthesia type, use of tranexamic acid, tourniquet, intraoperative blood loss, blood transfusion, central venous catheterization, and postoperative blood transfusion. Anticoagulation treatment included preoperative anticoagulation (the time from trauma to anticoagulation, time to start anticoagulation after admission, whether to interrupt, time to stop anticoagulation) and postoperative anticoagulation (time to start anticoagulation, whether to interrupt). The delayed anticoagulation time was > 24 h from trauma to the beginning of anticoagulation. The laboratory indicators collected at admission, 1 day after operation, and 3 days after operation include c-reactive protein (CRP) level, serum creatinine (Crea) level, serum urea level, prothrombin time (PT) level, prothrombin time ratio (PTR) level, international normalized ratio (INR) level, thrombin original activity (PTA) level, activated partial thromboplastin time (APTT) level, thrombin time (TT) level, fibrinogen (Fbg) level, D-dimer (DD) level, fibrinogen degradation product (FDP) level, white blood cell (WBC) count, hemoglobin (Hb) level, platelet count (PLT), neutrophil percentage (NEUT%), lymphocyte percentage (LYM%), and lactic acid (lac) level.

### Statistical analysis

SPSS23.0 was used to perform all the tests (IBM, Armonk, NY, USA). The enumeration data were expressed as number and percentage (%) and were evaluated by chi-square or Fisher’s exact test. Shapiro-Wilk test was used to determine whether the measurement data were normally distributed. Independent sample *T* test was used for measurement data conforming to normal distribution, and the results were expressed as mean ± standard deviation (*x* ± *s*). Measurement data of non-normal distribution were tested by Mann-Whitney *U* test, and the results were expressed as median (quartile) [M (Q1, Q3)]. Variables with statistical significance in univariate analysis were included in multivariate logistic regression analysis to determine independent risk factors. The statistical test level was set as *p* < 0.05.

## Results

### Clinical characteristics of DVT

#### Incidence of DVT

Table 1 presents the incidence of perioperative DVT. The total incidence of DVT was 40.25% (229/569); 152 patients had thrombosis before surgery, of which 21 patients had thrombosis progressed after surgery; 77 patients had newly formed thrombus after operation. The average preoperative DVT formation time was 6.55 ± 0.47 days (1–30 days) after the trauma and 6.67 ± 0.48 days (2–28 days) after the surgery.

**Table 1:** Perioperative incidence of deep vein thrombosis in patients with femoral and pelvic fractures [cases (%)]

Time of formation		Lower limb position			Fracture site				
Pre-operation	Post-operation	Affected side	Opposite side	Both sides	Femoral neck	Femoral intertrochanteric	Pelvis	Acetabulum	Femoral shaft
152 (26.71)	98 (17.22)	138 (60.26)	22 (9.61)	69 (30.13)	77 (30.92)	73 (43.98)	29 (43.28)	13 (48.14)	37 (61.67)

#### Dynamic changes of DVT types during perioperative period

**Fig. 1:**
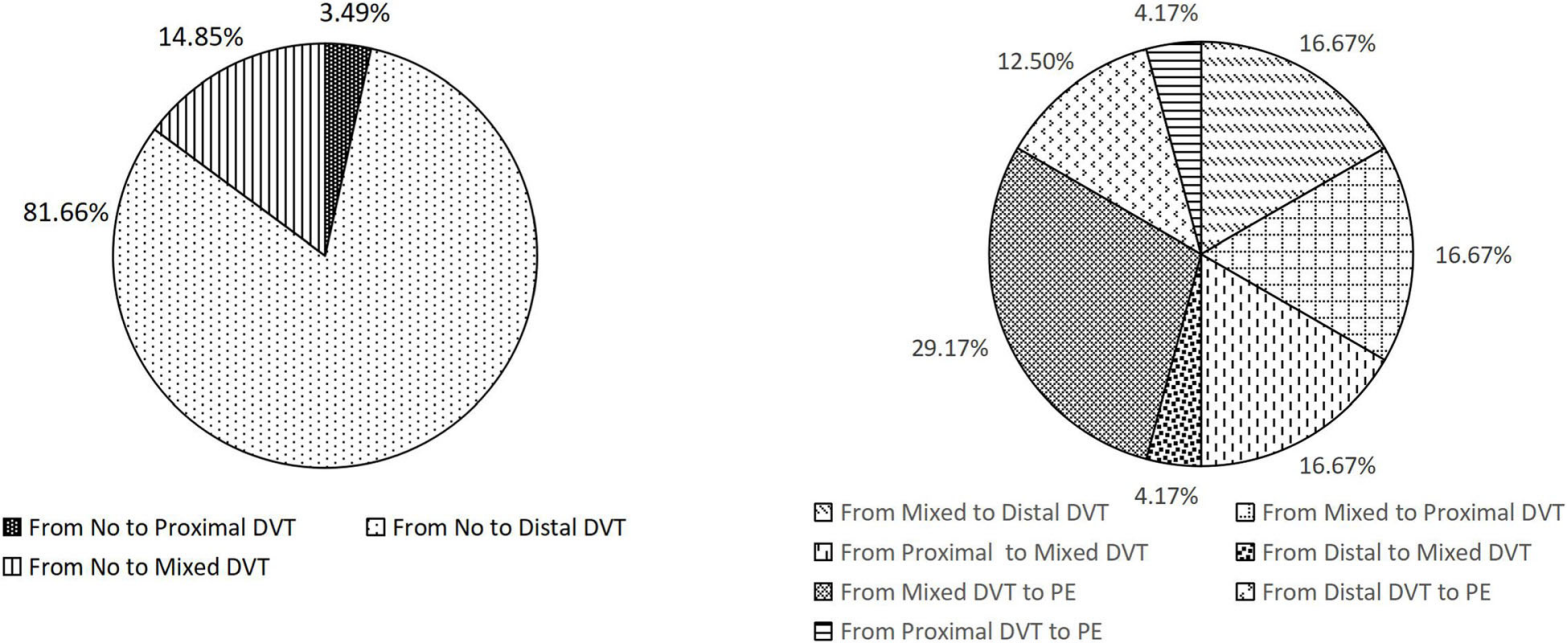
Dynamic changes of DVT types during perioperative period

The perioperative changes of DVT types in 229 patients are shown in Fig. 1. The incidence of distal, proximal, and mixed thrombus was 14.85%, 3.49%, and 81.66%, respectively. Among the 11 PE patients, 54.55% (6/11) complicated with distal thrombus, 18.18% (2/11) complicated with proximal thrombus, and 27.27% (3/11) complicated with mixed thrombus. The thrombosis type changed in 11.21% (13/116) of the 116 patients with intercalf muscle vein thrombosis.

### Preoperative DVT risk in patients with femoral and pelvic fractures

**Fig. 2:**
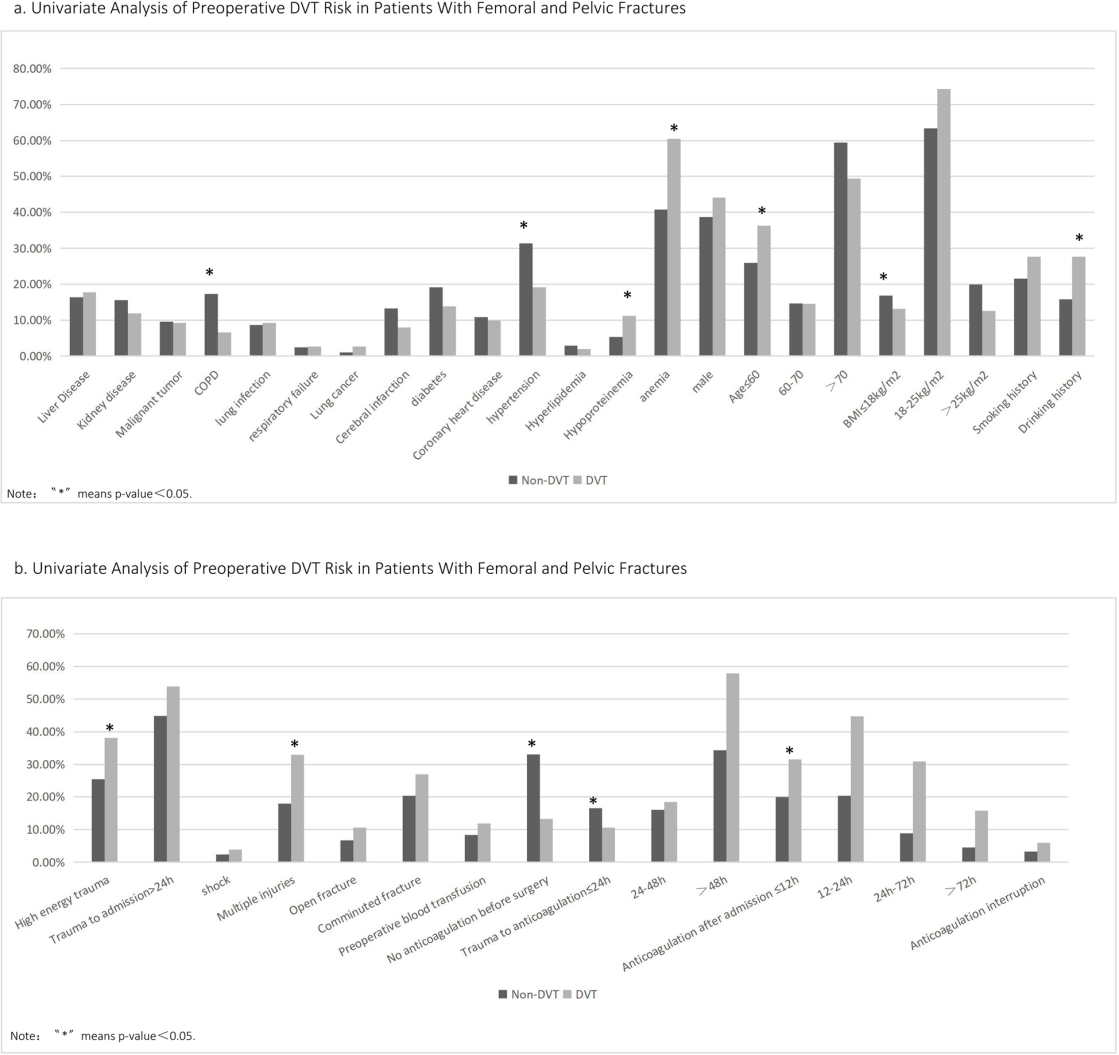
**a**, **b** Univariate analysis of preoperative DVT risk in patients with femoral and pelvic fractures

The preoperative DVT incidence rate was 26.71% (152/569). The univariate analysis results of general conditions, trauma, surgery, and anticoagulant protocols of patients in the non-DVT group and DVT group are shown in Fig. 2a, b. The univariate analysis results of the laboratory indexes are shown in Table 2. The results showed that there were no significant differences between the two groups in gender, smoking history, preoperative blood transfusion, open fracture, shock, liver disease, lymphocyte percentage, etc. (*P* > 0.05). Compared with the non-DVT group, there were statistically significant differences in preoperative non-anticoagulation (*P* = 0.000), multiple injuries (*P* = 0.000), high-energy trauma (*P* = 0.003), age > 60 years old (*P* = 0.047), BMI > 18 kg/m^2^ (*P* = 0.041), and drinking history (*P* = 0.001).

**Table 2:** Univariate analysis of preoperative DVT risk in patients with femoral and pelvic fractures

Influencing factor	Non-DVT	DVT	z/t	*P*
CRP (μg/L)	27.60 (13.50, 65.10)	27.80 (12.30, 58.60)	− 0.578	0.563
Urea (mmol/L)	6.10 (4.90, 8.00)	7.05 (5.03, 51.00)	− 4.192	0.000
Crea (μmol/L)	67 (56, 81)	72 (52, 83)	− 6.143	0.000
Lac (mmol/L)	2.20 (1.40, 2.40)	2.20 (1.43, 2.48)	− 1.202	0.229
WBC (10^9/L)	8.01 (6.35, 9.80)	8.01 (6.36, 10.22)	− 0.346	0.729
NEUT% (%)	74.58 ± 11.48	53.80 ± 12.42	7.726	0.000
LYM% (%)	15.00 ± 6.88	14.91 ± 5.63	0.256	0.798
PLT (10^9/L)	179.74 ± 92.02	190.13 ± 74.87	− 1.249	0.213
PT(s)	13.65 ± 1.47	11.09 ± 3.94	7.841	0.000
PTR	1.05 ± 0.11	5.14 ± 1.95	− 8.452	0.000
INR	1.06 ± 0.15	1.06 ± 0.09	0.490	0.625
PTA (%)	95.00 (87.00, 102.00)	86.50 (1.10, 98.00)	− 6.303	0.000
APTT(s)	37.48 ± 6.11	55.63 ± 27.14	− 8.169	0.000
TT(s)	16.27 ± 4.63	22.83 ± 11.05	− 7.101	0.000
Fbg (g/L)	4.03 ± 1.44	7.97 ± 2.84	− 8.222	0.000
FDP (μg/L)	13.60 (7.60, 31.45)	10.40 (4.27, 26.63)	− 3.480	0.001
D-D (mg/L)	5.17 (2.41, 12.06)	9.59 (5.03, 21.08)	− 5.412	0.000

**Fig. 3:**
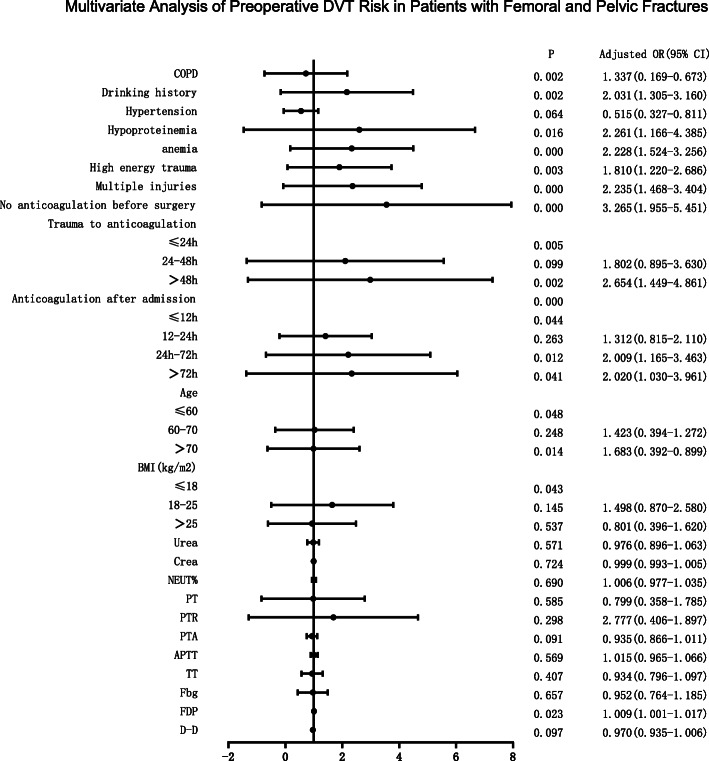
Multivariate analysis of preoperative DVT risk in patients with femoral and pelvic fractures

Multivariate logistic regression analysis was performed on variables with *P* < 0.05 (Fig. 3), and the results showed that preoperative non-anticoagulation, time from trauma to anticoagulation, anticoagulation after admission, COPD, hypoproteinemia, anemia, high-energy trauma, drinking history, advanced age, and elevated FDP level were independent risk factors for preoperative DVT. In terms of the risk of DVT before surgery, patients who started anticoagulation 12–24 h, 24–72 h, and > 72 h after admission were 1.31 times, 2.01 times, and 2.02 times the risk of DVT who started anticoagulation within 12 h of admission. The risk of preoperative DVT in patients with delayed anticoagulation at 24 h and 48 h after trauma was 1.80 and 2.65 times that of patients with early anticoagulation, respectively.

### Postoperative DVT risk in patients with femoral and pelvic fractures

**Fig. 4:**
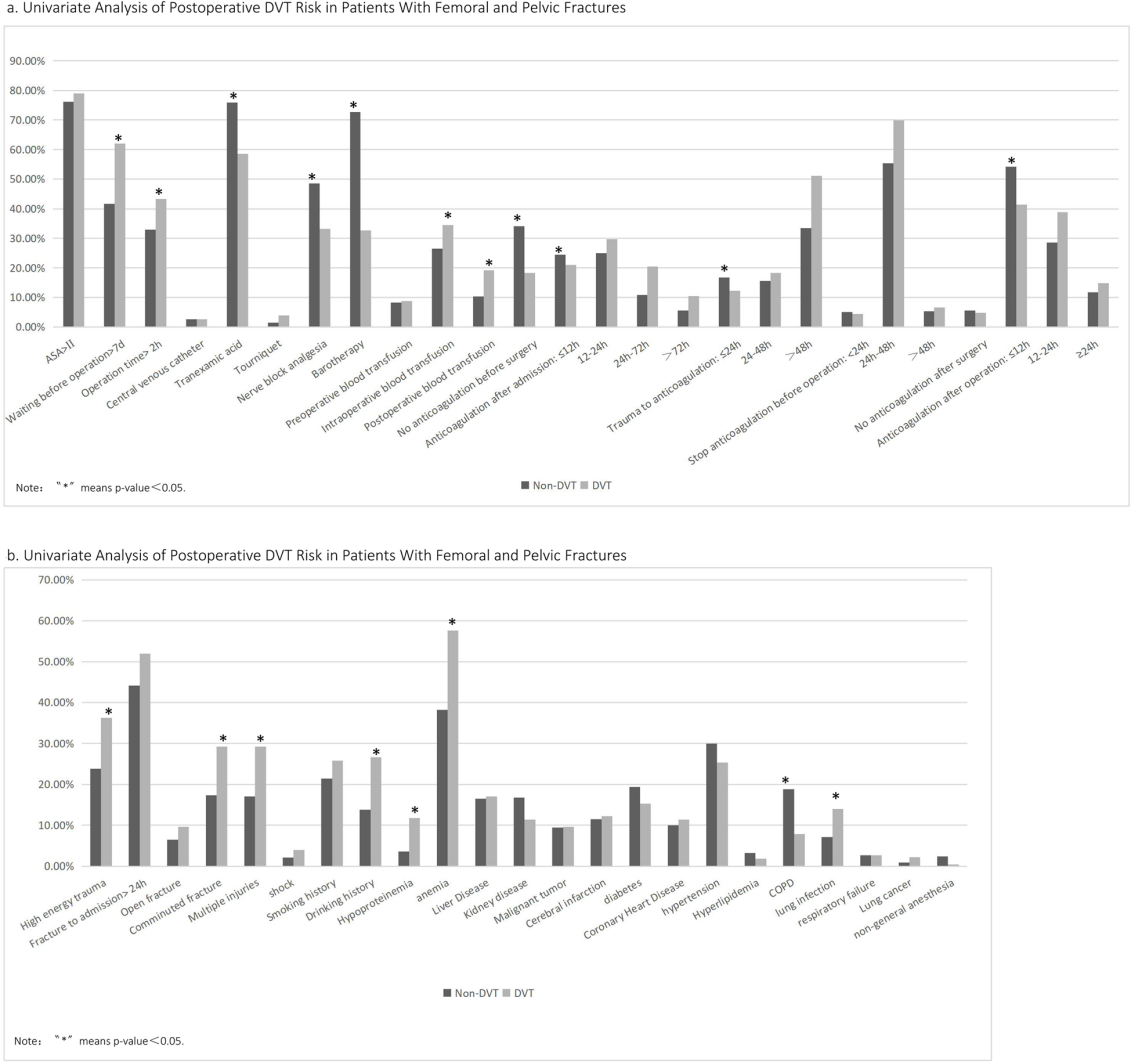
**a**, **b** Univariate analysis of postoperative DVT risk in patients with femoral and pelvic fractures

**Fig. 5:**
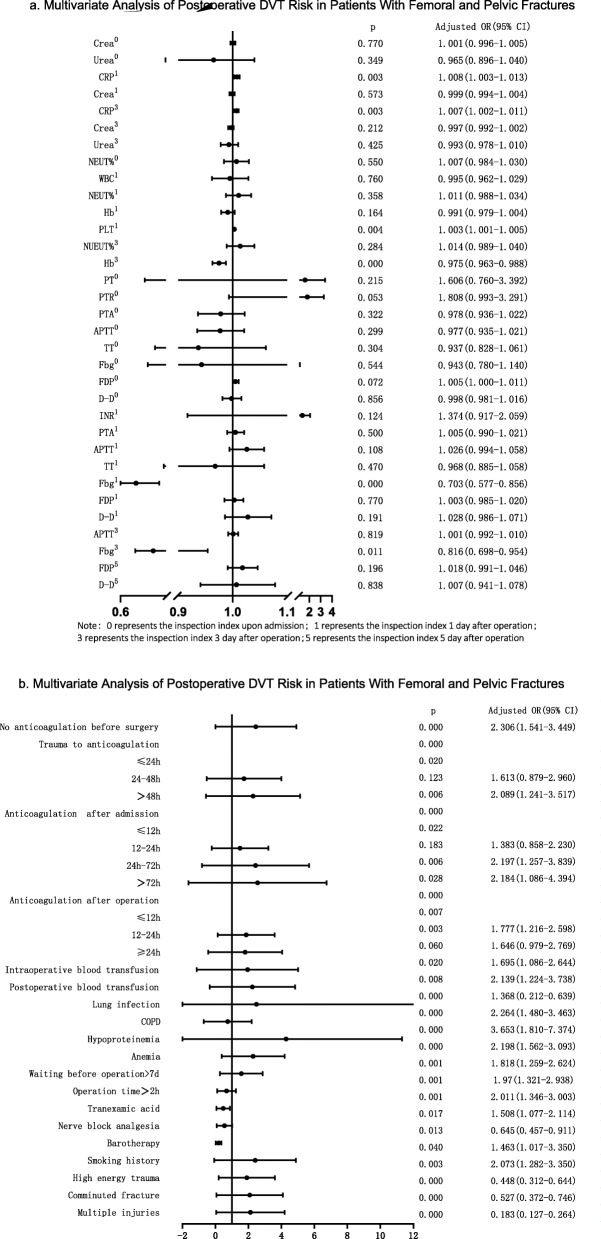
**a**, **b** Multivariate analysis of postoperative DVT Risk in patients with femoral and pelvic fractures

The incidence of postoperative DVT was 17.22% (98/569). 98.59% (561/569) patients underwent general anesthesia. Epidural anesthesia was performed in 0.88% (5/569) of the patients, and nerve block was performed in 0.53% (3/569) of the patients. The univariate analysis results between the two groups included comorbidities, trauma (Fig. 4a, b), blood routine, biochemical indicators (Table 3), and coagulation indicators (Table 3). Multivariate logistic regression analysis results (Fig. 5a, b) showed that delayed anticoagulation, lung infection, COPD, waiting for more than 7 days before surgery, anemia, hypoproteinemia, high energy trauma, multiple injuries, comminuted fractures, history of drinking, CRP and Fbg levels on the first day after surgery, HB, and CRP and Fbg levels on the 3rd day after surgery were independent risk factors for DVT in patients with femoral and pelvic fractures (*P* < 0.05). The risk of DVT in patients with intraoperative blood transfusion and postoperative blood transfusion increased to 1.46 times and 2.07 times, respectively. In terms of the risk of DVT after surgery, patients who started anticoagulation 12–24 h, 24–72 h, and > 72 h after admission were 1.38 times, 2.20 times, and 2.18 times that of patients who started anticoagulation within 12 h of admission. The risk of postoperative DVT in patients with delayed anticoagulation at 24 h and 48 h after trauma was 1.61 times and 2.09 times that of patients with early anticoagulation, respectively. The use of nerve block analgesia, tranexamic acid, and barotherapy were protective factors.

**Table 3:** Univariate analysis of postoperative DVT risk in patients with femoral and pelvic fractures

Influencing factor	Non-DVT	DVT	*Z*/*t*	*P*
CRP^a^ (μg/L)	26.70 (13.11, 62.60)	28.10 (12.57, 66.21)	− 0.428	0.668
Crea^a^ (μmol/L)	69.00 (55.81, 81.10)	60.01 (44.01, 76.49)	− 5.245	0.000
Urea^a^ (mmol/L)	6.10 (4.90, 8.00)	6.70 (4.90, 13.20)	− 3.063	0.002
Lac^a^ (mmol/L)	2.20 (1.40, 2.40)	2.20 (1.40, 2.40)	− 1.495	0.135
CRP^b^ (μg/L)	48.00 (27.30, 78.10)	56.80 (35.13, 86.18)	− 2.682	0.007
Crea^b^ (μmol/L)	66 (53, 80)	59 (51, 76)	− 2.615	0.009
Urea^b^ (mmol/L)	7.00 (5.40, 10.00)	6.70 (5.25, 9.05)	− 1.087	0.277
CRP^c^ (μg/L)	75.90 (40.10,105.00)	85.50 (50.65, 111.75)	− 2.518	0.012
Crea^c^ (μmol/L)	63.50 (51.00,77.80)	57.50 (47.00, 73.00)	− 2.643	0.008
Urea^c^ (mmol/L)	6.40 (4.80, 9.20)	5.90 (4,30, 8.58)	− 2.013	0.044
WBC^a^ (10^9/L)	8.03 (6.50, 9.72)	7.94 (6.05, 10.31)	− 0.929	0.353
NEUT%^a^ (%)	74.39 ± 11.16	61.06 ± 29.24	6.587	0.000
LYM%^a^ (%)	14.40 (10.80, 18.60)	13.20 (9.55, 18.20)	− 1.713	0.087
PLT^a^ (10^9/L)	180.89 ± 75.84	184.92 ± 85.58	− 0.590	0.555
WBC^b^ (10^9/L)	80.79 ± 10.05	10.02 ± 3.32	120.219	0.000
NEUT%^b^ (%)	99.88 ± 16.29	80.62 ± 13.31	15.438	0.000
Hb^b^ (g/L)	214.84 ± 88.58	93.95 ± 15.64	24.569	0.000
LYM%^b^ (%)	9.40 (6.20,12.60)	8.85 (5.83, 12.48)	− 1.083	0.279
PLT^b^ (10^9/L)	196.10 (151.60, 258.20)	213.00 (164.50, 285.50)	− 2.533	0.011
WBC^c^ (10^9/L)	7.94 ± 2.41	8.07 ± 2.76	− 0.575	0.566
NEUT%^c^ (%)	72.96 ± 11.79	74.90 ± 10.67	− 1.990	0.047
Hb^c^ (g/L)	96.53 ± 15.98	91.08 ± 14.19	4.169	0.000
LYM%^c^ (%)	14.58 ± 5.76	13.72 ± 6.28	1.678	0.094
PLT^c^ (10^9/L)	238.27 ± 97.23	244.81 ± 105.67	− 0.760	0.448
PT^a^ (s)	13.65 ± 1.51	11.96 ± 3.51	6.881	0.000
PTR^a^	1.03 (0.99, 1.08)	1.05 (1.01, 1.22)	− 5.110	0.000
INR^a^	1.04 (0.99, 1.09)	1.04 (0.99, 1.10)	− 0.666	0.505
PTA^a^ (%)	95.10 (87.10, 101.20)	89.10 (69.25, 99.10)	− 5.133	0.000
APTT^a^ (s)	37.72 ± 6.27	49.19 ± 24.07	− 7.054	0.000
TT^a^ (s)	16.36 ± 4.98	20.44 ± 9.67	− 5.871	0.000
Fbg^a^ (g/L)	3.95 (3.19, 4.74)	4.28 (3.28, 6.66)	− 3.780	0.000
FDP^a^ (μg/L)	13.65 (7.90, 32.75)	12.20 (5.15, 25.45)	− 3.132	0.002
D-D^a^ (mg/L)	5.31 (2.51, 12.37)	7.81 (3.70, 17.86)	− 3.393	0.001
PT^b^ (s)	13.80 (13.20, 14.30)	13.90 (13.30, 14.60)	− 1.696	0.090
PTR^b^	1.05 (1.02, 1.09)	1.06 (1.02, 1.11)	− 1.644	0.100
INR^b^	1.09 ± 0.27	1.10 ± 0.13	120.370	0.000
PTA^b^ (%)	39.53 ± 7.94	87.95 ± 15.83	− 42.716	0.000
APTT^b^ (s)	16.09 ± 2.66	40.14 ± 9.07	− 38.911	0.000
TT^b^(s)	4.63 ± 1.46	15.89 ± 2.98	− 53.004	0.000
Fbg^b^ (g/L)	4.51 (3.83, 5.22)	4.27 (3.55, 4.91)	− 2.951	0.003
FDP^b^ (μg/L)	4.60 (4.40, 16.10)	12.30 (6.25, 22.45)	− 3.842	0.000
D-D^b^ (mg/L)	3.31 (1.42, 6.65)	4.48 (2.15, 7.98)	− 3.730	0.000
PT^c^ (s)	14.01 ± 6.45	13.62 ± 1.21	0.895	0.371
PTR^c^	1.03 (0.99, 1.08)	1.04 (0.99, 1.08)	− 0.685	0.494
INR^c^	1.05 ± 0.12	1.06 ± 0.11	− 0.458	0.647
PTA^c^ (%)	93.41 ± 16.69	93.29 ± 15.24	0.090	0.928
APTT^c^ (s)	40.00 (36.60, 45.00)	42.10 (37.10, 47.10)	− 2.629	0.009
TT^c^ (s)	16.07 ± 7.34	16.25 ± 9.34	− 0.254	0.800
Fbg^c^ (g/L)	5.54 ± 1.57	5.27 ± 1.55	2.093	0.037
FDP^c^ (μg/L)	9.70 (5.90, 15.00)	10.45 (6.57, 15.53)	− 1.391	0.164
D-D^c^ (mg/L)	2.98 (1.70, 5.40)	3.37 (2.11, 5.76)	− 1.805	0.071
PT^d^ (s)	13.30 (12.90, 13.90)	13.40 (12.70, 13.90)	− 0.659	0.510
PTR^d^	1.02 (0.99, 1.07)	1.02 (0.98, 1.07)	− 0.261	0.794
INR^d^	1.03 (0.99, 1.10)	1.02 (0.97, 1.09)	− 0.886	0.376
PTA^d^ (%)	90.53 ± 23.31	93.55 ± 20.07	− 0.346	0.135
APTT^d^ (s)	40.10 (35.03, 44.00)	39.70 (35.90, 43.56)	− 0.106	0.916
TT^d^ (s)	15.50 (15.00, 16.50)	15.60 (14.80, 16.60)	− 0.405	0.686
Fbg^d^ (g/L)	5.23 ± 1.90	5.26 ± 1.87	− 0.171	0.864
FDP^d^ (μg/L)	11.00 (7.19, 16.38)	12.90 (8.50, 22.0)	− 3.879	0.000
D-D^d^ (mg/L)	4.01 (2.39, 7.06)	5.13 (3.14, 8.37)	− 3.884	0.000

^a^The inspection index upon admission

^b^The inspection index 1 day after operation

^c^The inspection index 3 days after operation

^d^The inspection index 5 days after operation

## Discussion

In recent years, many literatures have reported the incidence of perioperative DVT in fracture patients, and there are differences in the incidence of DVT in different regions, races, and populations. The incidence of VTE after total knee arthroplasty was 20.67% in the USA after aspirin and barotherapy with multimodal prophylaxis [3]. Yukizawa et al.’s prospective, single-center study reported that the preoperative and postoperative VTE rates of 419 total hip replacement patients in Japan were 11.4% and 10.5%, respectively [4]. However, a retrospective study of 1825 lower limb fractures in China found that the preoperative and postoperative DVT rates were 30.0% and 43.4%, respectively [5]. The results showed that the incidence of DVT before and after femoral and pelvic fractures was 26.71% and 17.22%, respectively. Currently, the incidence of DVT in fracture patients has not been significantly reduced by “more aggressive” anticoagulant regimens.

Drug prevention is the main measure for the prevention and treatment of DVT, but there are controversies about the indications, drug selection, starting and ending time, safety, and effectiveness of anticoagulation. The results of this study showed that the risk of DVT increased to 2.31-fold in patients with femoral and pelvic fractures without anticoagulation before surgery. Contrary to Liu et al.’s conclusion, preoperative anticoagulation in patients with hip fractures does not significantly reduce the risk of postoperative DVT or PE [6]. Haut et al. found that approximately 50% of VTE occurred in patients receiving “optimal prevention” [7]. Many problems of anticoagulation are still inconclusive, but it is still advocated that anticoagulation should be actively used in patients with high risk of perioperative bone trauma who have no anticoagulation contraindications. LMWH is now recommended as the preferred anticoagulant drug for major orthopedic surgery. For the timing of prophylactic anticoagulation, ACCP recommends the use of LMWH anticoagulation at ≥ 12 h before surgery or ≥ 12 h after surgery [8]. BCSH recommends starting LMWH approximately 12 h before surgery or 6 to 8 h after surgery [9]. However, ASH regards 12 h postoperatively as the demarcation point between early and late postoperative anticoagulation [10]. Although the start time of anticoagulation in the guidelines is still controversial, it was mentioned 12 h before or 12 h after surgery. Weng et al.’s investigation on ERAS and thromboprophylaxis in Chinese artificial joint replacement surgery found that among orthopedic doctors 22.8% (342/1497), 31.9% (478/1497), and 37.7% (564/1497) chose to start anticoagulation within 6 h, 6–12 h, and 12–24 h after surgery, respectively [11]. 72.23% (411/569) patients in our center started anticoagulation ≥ 12 h before surgery, and 51.76% (279/539) patients started anticoagulation within 12 h after surgery. This is consistent with the current status of perioperative anticoagulation in Chinese patients with bone trauma and also follows the recommendations of relevant guidelines. This study found that the risk of preoperative DVT for patients who started anticoagulation 12–24 h, 24–72 h, and > 72 h after admission was 1.31 times, 2.01 times, and 2.02 times that of anticoagulation within 12 h of admission. For the risk of postoperative DVT, this value was 1.38 times, 2.20 times, and 2.18 times, respectively. The early stage of trauma is hypercoagulable state, and early anticoagulation after admission can reduce the risk of DVT by eliminating contraindications. In addition, we also collected the time from trauma to anticoagulation. We found that patients with delayed anticoagulation beyond 24 h and 48 h after trauma were 1.80 times and 2.65 times the risk of preoperative DVT in early anticoagulation patients, and 1.61 and 2.09 times the risk of postoperative DVT. Our results were consistent with Xia et al. [12]. Delayed anticoagulation 24 h after trauma was positively correlated with the occurrence of VTE, and the correlation between the two was more significant when the delayed anticoagulation time exceeded 48 h. These lead us to think that delayed anticoagulation after trauma still exists under the “positive” concept of early anticoagulation. This might be related to the fact that most patients are transferred to our hospital from other hospitals, waiting for blood routine and coagulation function tests, and the definition of “contraindication” for anticoagulation by different doctors. Although the “optimal” timing of preventive anticoagulation needs to be further verified, how to minimize the interference of these controllable factors and realize anticoagulation as early as possible is a challenge. Interruption of preventive anticoagulation is common in trauma and in patients undergoing major surgery and is associated with patient rejection, surgical timing, and nursing errors. Louis et al. confirmed that anticoagulation interruption is an independent risk factor for DVT in trauma patients aged ≥ 50 years [13]. However, we found no correlation between anticoagulation interruption and DVT, which may be related to the risk of bleeding associated with anticoagulation interruption in this study. For patients hospitalized for total knee and hip replacement within 5 days, Samama et al., Petersen et al., and other large-sample, multi-center studies have confirmed that anticoagulation in these patients is safe only in the hospital [14, 15]. However, there are few studies on the timing of anticoagulation after surgery. This study found that 12–24 h and 24 h after surgery were 1.78 times and 1.65 times of the risk of DVT in patients who started anticoagulation within 12 h after surgery, respectively. Therefore, starting anticoagulation within 12 h after surgery can reduce the risk of DVT, but the benefits of early anticoagulation after surgery should be weighed against the increased risk of bleeding.

Among fracture patients, the current routine anticoagulant regimens are used for the prevention and treatment of DVT in patients with different risks, which does not change the current situation that the incidence of DVT is still relatively high. It is particularly important to further clarify independent risk factors for DVT to identify high-risk groups. The occurrence of DVT was significantly correlated with the fracture site. Similar to the results of Adam et al. and Zhang et al. [16, 17], the incidence of hip fracture (74.90%) was relatively high. Considering that most patients with hip fractures may have three risk factors of Virchow at the same time  [18], we should pay attention to the screening of DVT in these patients with fractures at specific sites and formulate reasonable individualized prevention and treatment strategies. Meizoso et al. used RAP score in a retrospective cohort study of 1233 trauma patients and found that transfusion of more than 4 units was an independent risk factor for DVT [19]. The preoperative, intraoperative, and postoperative blood transfusion rates of 569 patients in this study were 8.44% (48/569), 29.70% (169/569), and 13.88% (79/569), respectively. It was further found that intraoperative blood transfusion and postoperative blood transfusion were independent risk factors for postoperative DVT, while preoperative blood transfusion was not correlated with DVT. Perhaps we should reconsider the indications and necessity of blood transfusion in practice. Song et al. reported that most patients with postoperative DVT had complicated DVT before surgery [20]. In this study, preoperative DVT occurred in 26.71% of the patients, which was considered to be related to the longer average preoperative waiting time. Most of the current recommendations are that surgery should be performed as soon as possible within 48 h after trauma to reduce the incidence of VTE [21]. But in practice, 66% of patients with hip fractures delayed surgery [22]. The reasons for delayed surgery may be as follows: 67.66% (385/569) of the patients were transferred to our hospital from other hospitals due to serious illness or limited medical conditions. Preoperative preparation for patients with medical diseases and routine preoperative examinations such as DVT screening will also delay surgery. Recently, Luksameearunothai et al. confirmed that Caprini score ≥ 12 points should be used for preoperative ultrasound examination in elderly patients with hip fracture, and patients with Wells score ≤ 1 point can be safely operated immediately [23]. There is a conflict between adequate preoperative preparation and early surgery, and how to achieve a comprehensive and scientific evaluation of severe patients is the key to avoid unnecessary delay in surgery. Shahi et al. and Parvizi et al. reported that chronic pulmonary disease is associated with postoperative DVT in patients with fracture [24, 25]. We further found that COPD was an independent risk factor for preoperative DVT and pulmonary infection, and COPD was an independent risk factor for postoperative DVT. Systemic inflammation, hypoxemia, oxidative stress, endothelial dysfunction, and prethrombotic status would increase the risk of VTE in patients with COPD [26]. Elderly fracture patients with COPD may be chronically hypoxic for a long time. Lying in bed and immobilization after a fracture would increase the risk of pulmonary infection, as well as limited mobility and lack of muscle pumping, leading to venous stasis and hypercoagulability in the lower extremities [27]. Therefore, active prevention and treatment of pulmonary diseases in patients with fractures may reduce the incidence of perioperative DVT. The European Guidelines for the prevention of perioperative VTE indicate that correcting preoperative anemia could reduce the incidence of postoperative VTE in elderly patients [28]. Both we and Feng et al. found that preoperative anemia was an independent risk factor for perioperative DVT in fracture patients [29], which may be associated with increased D-dimer caused by anemia [30]. C-reactive protein levels on the first and third days after surgery were independent risk factors for postoperative DVT, and acute inflammation reflected by high levels of C-reactive protein was considered as the trigger factor for VTE [31]. The timing and necessity of perioperative anti-infective therapy need to be further validated. Hypoproteinemia is another independent risk factor for DVT. It may be that the swelling of the lower extremities leads to weakened muscle pumping and slow blood flow, and the pain caused by swelling also reduces the active activity of the lower extremities. Many studies have confirmed that hypertension, hyperlipidemia, diabetes, and other chronic diseases were risk factors for DVT. This result was not obtained in this study, which may be related to the small sample size and missed diagnosis of doctors in this single center study. Drinking history, advanced age, high-energy trauma, multiple injuries, and comminuted fracture are independent risk factors for DVT. Although these factors cannot be optimized, they are helpful for early identification of high-risk groups. D-dimer detection with high sensitivity and low specificity is mostly used to exclude patients with suspected VTE, and its increased level is related to the degree of trauma, fracture site, degree of inflammatory response, pregnancy, etc. In this study, although the increase of D-dimer was not found to be an independent risk factor for DVT, the postoperative D-dimer level of the DVT group was significantly higher than that of the non-DVT group, and the D-dimer test of almost all patients was positive. This is consistent with Wang et al.’s view that D-dimer detection has poor specificity in trauma patients and its significance is limited [32]. Neither Tyagi et al. nor we found the correlation between intraoperative tourniquet use and DVT, considering that short duration of tourniquet use could not significantly affect the blood status of lower extremities. But Tyagi et al. still believes that patients with higher VTE risk should consider using a tourniquet during surgery [33]. Whether this simple, low-cost, low-risk intraoperative method can reduce the risk of postoperative DVT remains to be further studied. The results showed that the average preoperative DVT formation was 6.55 ± 0.47 days after the trauma and 6.67 ± 0.48 days after the surgery, which may be related to the peak time of blood hypercoagulability. The D-dimer level reached its peak on the 1st and 7th days after the fracture surgery [34]. This study reviewed the changes in the types of DVT during the perioperative period, 54.55% (6/11) complicated with distal thrombosis, 18.18% (2/11) complicated with proximal thrombosis, and 27.27% (3/11) complicated with mixed thrombosis. The incidence of distal thrombus progression to PE is relatively high, so thrombus type should not be our sole criterion for screening high-risk populations. The therapeutic anticoagulation of intermuscular vein thrombosis of the calf is controversial. Recent studies have shown that the absence of preoperative anticoagulation does not increase the risk of progression of intermuscular vein thrombosis in the calf and may aggravate postoperative anemia [12]. In this study, 11.21% of the intermuscular venous thrombosis of the calf showed progress, and it seems that therapeutic anticoagulation is necessary for such patients.

Venography is the gold standard for the diagnosis of VTE, but its application is limited due to its invasiveness, unrepeatability, and possible renal and venous injuries caused by contrast agents. Noninvasive and repeatable ultrasound examination has been widely used in clinical practice in recent years. Abe et al.’s study confirmed that the dilatation of plantar vein during ultrasound examination is an independent predictor of DVT after major orthopedic surgery [35]. However, lower extremity ultrasound examination is not suitable for patients with partial fractures. For example, patients with hip fracture cannot change the body position examination, and it is difficult to form a clear image for those with severe limb swelling and thick surgical area covered by dressings. The diagnostic examination of DVT is mainly to avoid the occurrence of fatal VTE, but how to identify early and prevent the occurrence of DVT is a more important goal. It is valuable to analyze the risk factors of DVT during the whole perioperative period for femoral and pelvic fractures.

We analyzed the correlation between anticoagulation regimen, perioperative blood transfusion, pulmonary disease, and other perioperative influencing factors and DVT. However, this study has the following limitations: Firstly, this is a single-center, retrospective case-control study, which is prone to confounding factors. For example, the influencing factors not included in the study may be potential risk factors for DVT, and the study subjects were not operated by the same surgeon. Secondly, only color Doppler ultrasonography was used to determine DVT, and potential DVT patients with clinical symptoms such as lower limb swelling, pain, and elevated skin temperature were not included in the case group, which reduced the effectiveness of the result data. Thirdly, all patients received lower extremity venous ultrasound examination after admission. However, there may be a deviation between DVT formation time and ultrasonic examination time in some patients with delayed admission. Fourthly, this study only reviewed the clinical medical records of the patients during their hospitalization and did not follow-up the patients after discharge, which may have omitted the occurrence of DVT after discharge, thus underestimating the incidence of DVT.

## Conclusion

In summary, the current anticoagulation and other DVT prevention and treatment programs have reduced the incidence of DVT of femoral and pelvic fractures but have not changed the current situation of high incidence of DVT. By analyzing the risk factors of DVT throughout the perioperative period, optimizing the perioperative blood transfusion, preoperative lung disease, hypoproteinemia, anemia, inflammation, etc., and early surgery within 7 days after trauma may further reduce its incidence.

## Data Availability

All the data will be available upon motivated request to the corresponding author of the present paper.

## Abbreviations

VTE: Venous thromboembolism
DVT: Deep vein thrombosis
PE: Pulmonary embolism
COPD: Chronic obstructive pulmonary disease
BMI: Body mass index
ASA: American Society of Anesthesiologists
CRP: C-reactive protein
Crea: Creatinine
PT: Prothrombin time
PTR: Prothrombin time ratio
INR: International normalized ratio
PTA: Thrombin original activity
APTT: Activated partial thromboplastin time
TT: Thrombin time
Fbg: Fibrinogen
DD: D-dimer
FDP: Fibrinogen degradation products
WBC: White blood cell
Hb: Hemoglobin
PLT: Platelet count
NEUT%: Neutrophil percentage
LYM%: Lymphocyte percentage
Lac: Lactic acid
